# A Non-Label and Enzyme-Free Sensitive Detection Method for Thrombin Based on Simulation-Assisted DNA Assembly

**DOI:** 10.3390/s18072179

**Published:** 2018-07-06

**Authors:** Yingying Zhang, Luhui Wang, Yanan Wang, Yafei Dong

**Affiliations:** 1 School of Computer Science, Shaanxi Normal University, Xi’an 710119, China; zhangyingying@snnu.edu.cn (Y.Z.); wangyanan@snnu.edu.cn (Y.W.); 2 College of Life Sciences, Shaanxi Normal University, Xi’an 710119, China; wangluhui@snnu.edu.cn; 3 National Engineering Laboratory for Resource Developing of Endangered Chinese Crude Drugs in Northwest of China, Shaanxi Normal University, Xi’an 710119, China

**Keywords:** thrombin detection, fluorescence, colorimetric, isothermal cycling signal amplification, computation and simulation

## Abstract

Taking advantage of the high selectivity of aptamers and enzyme-free catalyzed hairpin assembly (CHA) amplification strategy, we herein describe a label-free and enzyme-free sensitive fluorescent and colorimetric strategy for thrombin detection in this paper. In the presence of target, the corresponding aptamer of the partial dsDNA probes will bind to the target and liberate the initiation strand, which is artfully designed as the “on” switch for hairpin assembly. Moreover, the displaced initiation strand partakes in a multi-cycle process and produces numerous G-quadruplexes, which have a remarkable enhancement in fluorescent/colorimetric signal from NMM (*N*-methyl-mesoporphyrin IX) and TMB (3,3′,5,5′-tetramethylbenzidine), respectively. The proposed amplification strategy for thrombin detection is of high sensitivity, down to 2.4 pM, and also achieves colorimetric signals that are able to be distinguished by naked eye. More importantly, the thermodynamics of interacting DNA strands used in our work, and the process of toehold strand displacement-driven assembly are simulated before biological testing, verifying the feasibility theoretically, and simplifying the subsequent actual experiments. Therefore, our approach and simulation have a certain potential application in biomarker detection and quantitatively monitor for disease diagnosis.

## 1. Introduction

Biosensors are of critical importance for detection of bioactive molecules from a biomedical, environmental, and security point of view [1,2]. Biomolecules related to the living activity in nature, such as genes, proteins, and ions, have attracted growing attention in cancer diagnosis and biomedicine. Some special proteins have a decisive function in the early stages of diagnosing a disease and pathological state as the molecular mechanism of life [3]. Especially, thrombin, a pivotal enzyme for all major thrombotic processes, plays an indispensable role in hemostasis, thrombosis, inflammation, and vascular remodeling [4]. Accordingly, a series of methods for thrombin detection have been developed in recent years, due to the important biological function of the protein [5,6,7,8,9,10]. Though enzyme-linked immunosorbent assay (ELISA) has found widespread use in detecting protein [11,12], we have to admit that the antibody-based assay is time-consuming, because it involves incubation, reaction, and multi-step washing [13,14]. In order to reduce costs and simplify operations as much as possible, non-labeled approaches for protein detection have attracted increasing interest [5,6]. However, what cannot be ignored is that a majority have no advantages in sensitivity and dynamic detection range.

To overcome this limitation of sensitivity, a variety of signal amplification techniques have been put forward to study thrombin detection and medical diagnosis, including nicking enzyme-assisted fluorescent signal enhancement [7,8], aptamer-based hybridization chain reaction [15], magnetic microparticle-based sandwich assays [9,10], and so on. Unfortunately, the analytical strategies based on enzymatic action and conjugation of aptamers with dye marker will lead to new problems, while these methods can largely improve the low sensitivity. For example, the binding affinity between modified aptamers and molecular target may be different from the previous one [16]. In addition, the activity of protein enzymes also depends, to a large extent, on the reaction conditions [17]. For these reasons, it is extremely necessary to develop various signal amplification methods for the detection of small molecules without involving of enzymes or modification of aptamers.

Catalytic hairpin assembly (CHA) is an isothermal, enzyme-free signal amplification reaction based on strand displacement reaction [18]. It has been extensively utilized to design sensitive biosensors for nucleic acids, proteins, and small molecules, due to many outstanding features, such as highly specific Watson–Crick base-pairing capability and excellent biocompatibility [7,17,19]. However, most of the CHA-based detection methods necessitate modification with fluorophore and quencher, which increases the overall cost and complexity of detection. Therefore, in the process of CHA reaction, we introduce a kind of special DNA structure, G-quadruplex, instead of modification. In recent years, their great potential in the design and application of innovative biosensing for molecular detection has been found [20,21,22]. The formation of special DNA structure would significantly enhance fluorescence under the action of the special cationic dyes, which is extensively used in development of biosensors [23,24]. For example, it will strongly interact with NMM, yielding a significantly enhanced fluorescence together [25,26]. On the other hand, the certain G-rich DNA sequences can work with the hemin to form peroxidase-mimicking DNAzymes, catalyzing the H_2_O_2_-mediated oxidation [27,28]. Therefore, the G-quadruplex/hemin enzyme has been adopted in various chemiluminescent, colorimetric, and fluorescent biosensors for the quantitative detection of biomolecules and the activity analysis of enzymes [29,30,31].

In fact, biological experiments usually have the disadvantages of complex operation, and being time-consuming and expensive, and computer technology can solve these problems, to some extent, by simulation. Among them, the full tertiary structure and the associated biological function of nucleotide sequences found in living organisms can be simulated and predicted by calculating the optimal free energy secondary structure [32]. A functional nucleic acid system can be constructed to execute dynamic mechanical tasks by properly designing the nucleotide sequence [33]. Consequently, in order to improve the efficiency and productivity of DNA device design and analysis, we innovatively combine simulation with biological experiments during the design and analysis process of the model [34].

On the basis of the above considerations, taking the advantages of the higher selectivity and affinity of aptamers [35] based on enzyme-free catalyzed hairpin assembly (CHA), we proposed a new enzyme-free, non-label sensitive fluorescence and colorimetric method to realize amplification detection of thrombin [36]. Our method can reach visible color change, and has the advantage of a low fluorescent detection limit, about $10^{-12}$ M. More importantly, the design can be visualized and analyzed by simulations before biological testing, to eliminate some negative consequences and simplify the subsequent actual experiments.

## 2. Experimental Details

### 2.1. Materials and Reagents

The DNA strands used in our experiments were purified from Sangon Biotech Co. (Shanghai, China), and their sequences are list in Table 1. Thrombin derived from the bovine plasma (lyophilized powder, 40–300 NIH units/mg protein) and 3,3′,5,5′-tetramethylbenzidine (TMB) were obtained from Sigma (St. Louis, MO, USA). *N*-methyl-mesoporphyrin IX (NMM) was purchased from J&K Scientific Ltd. (Beijing, China) and diluted with dimethyl sulfoxide (DMSO). This prepared solution was kept away from light at −20 °C before use. The fetal bovine serum samples were purchased from Lanzhou Roya Bio-Technology Co. (Lanzhou, China), Led, and diluted with reaction buffer 10 times before use. The others were purchased from Xi’an JingBo Bio-Technique Co. (Xi’an, China).

### 2.2. Apparatus

The fluorescence spectra can be measured using a fluorescent scanning spectrometer for NMM at 399 nm excitation and 610 nm emission by EnSpire ELIASA from PerkinElmer USA (Shanghai, China), which was also used for absorbance measurements for TMB at 450 nm emission. The gel image was obtained by Molecular Imager (Peiqing science & Technology. Co. Ltd., Shanghai, China).

### 2.3. Procedure for Thrombin Assay

H1, H2, and the mixed solution of aptamer/S strand (aptamer/S, 2 mM) were separately heated at 90 °C for 5 min, cooled down slowly to room temperature, and stored at 4 °C. Subsequently, thrombin, aptamer/S probe solution (50 nM) and working buffer were mixed for 30 min at 37 °C. Then, H1 (300 nM) and H2 (300 nM) were mixed with the solutions above, and the self-assembly process lasted for 120 min at 37 °C. After that, part of the product of CHA was combined with NMM (1.5 μM), followed by incubation for 25 min at room temperature, and the fluorescence intensity was recorded, finally. In addition, hemin (600 nM) was added into another part of solution above, and incubated at 25 °C. After 30 min, 490 μL TMB/H_2_O_2_ solution was mixed with 10 μL reaction product for 10 min. Then, 500 μL H_2_SO_4_ (2 M) was used to terminate this oxidation reaction, and the absorbance was recorded 5 min later.

### 2.4. Gel Electrophoresis Analysis

Different samples were detected by 10% native polyacrylamide gel using 6× loading buffer at a constant voltage of 80 V for 120 min in 1 × TBE buffer. After staining with EtBr for 10 min, the gel image can be observed with a molecular imager.

## 3. Results and Discussion

### 3.1. Principle of Thrombin Detection

**Figure 1:**
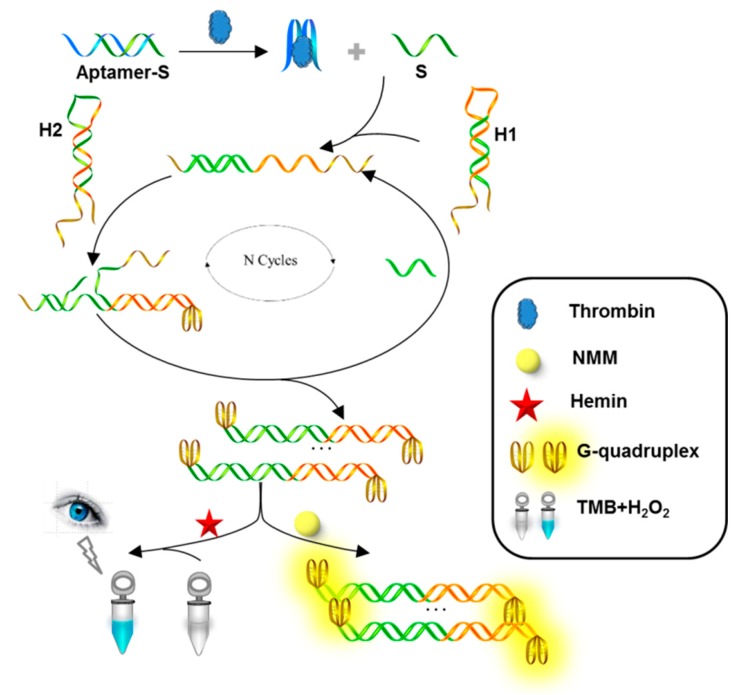
Schematic diagram of detecting platform based on hairpin assembly and special G-quadruplex structure.

The basic principle for non-label, enzyme-free optical protein detection platform is shown in Figure 1. The system consists of thrombin-aptamer recognition process, CHA, and signal readout. Specifically, a pair of ingenious hairpins DNA has been designed, containing stem as the substrate of initiation strand, and G-rich sequence as a quadruplex-forming oligomer. At the beginning of the reaction, thrombin binds to the aptamer strand, due to the high binding affinity with corresponding aptamer, and the S strand is free. The liberated S strand acts as a molecular switch to trigger strand displacement reaction in next CHA circulation, forming the duplex strands (H1:H2) carrying the G-quadruplexes at both ends. In particular, the displaced S strand partakes in a multi-cycle process again, leading to the generation of multiple G-quadruplex structures. Finally, the G-quadruplex configuration can interact with NMM to emit significantly enhanced fluorescent signal, or form G-quadruplex/hemin DNAzyme by the function of hemin, to catalyze the H_2_O_2_-mediated oxidation. By contrast, the self-assembly reaction of hairpins could not be carried out without thrombin, because of no trigger switch-S strand. Consequently, there was only small amounts of G-quadruplexes, and low fluorescence emission of NMM and weak colorimetric signal of TMB, in this case.

### 3.2. Simulation Experiment

In order to save time and lab resources as soon as possible, it is extremely necessary to simulate the method we have constructed in this paper before biological experiments. Therefore, we introduce an algorithm firstly for analyzing the thermodynamic properties of interacting DNA sequences to demonstrate the validity of DNA strands in our strategy. Here, thermodynamic models based on nucleic acid secondary structure and nearest-neighbor identities [37] underlying dynamic programming algorithms are employed in predicting the minimum free energy (MFE) of the secondary structure [38] and computing the partition function over secondary structure level [39]. Moreover, the partition function can be conducive for studying and evaluating the conformational ensembles, as well as the designed DNA/RNA sequences in the system.

**Figure 2:**
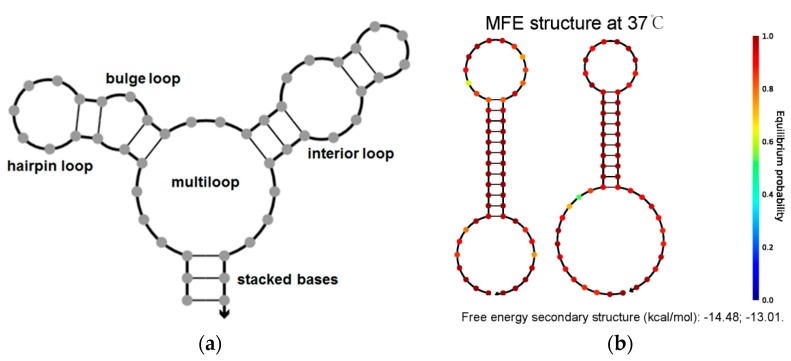
(**a**) Canonical loops of nucleic acid secondary structure; (**b**) The MFE structure of H1 and H2 by simulation.

Specifically, for a nucleic acid secondary structure, thermodynamic models are used to decompose the base-pairing graph for a structure into distinct loop configurations that are related to empirically measured enthalpic and entropic terms according to loop sequence, length, and type [40]. As shown in Figure 2a, the base-pairing graph is constructed in a variety of configurations, such as hairpin loops, stacked bases, an interior loop, a multiloop and a bulge loop. In brief, the free energy of secondary structure *s* can be calculated in Equation (1). The partition function in Equation (2) can be used to calculate the equilibrium probability of any secondary structures *s* contained in structural ensemble Γ (Equation (3)), where *T* and *k* are temperature and Boltzmann constant, respectively.

(1) $$\;\Delta G\left(s\right)\;=\;\underset{\mathrm{loop}\in s}{\sum }\Delta G\left(\mathrm{loop}\right)$$

(2) $$\;Q=\;\underset{s\in \Gamma }{\sum }e^{-\Delta G\left(s\right)/kT}$$

(3) $$\;p\left(s\right)=\;\frac{1}{Q}\;e^{-\Delta G\left(s\right)/kT}$$

Hence, the partition function and equilibrium probability for a given sequence can be computed according to above principle and algorithm. A CHA amplification device is composed of two hairpin structures (H1 and H2) that are designed to coexist under metastable state in the absence of “molecular switch”-S strand (Figure 2b). The MFE structure of H1 and H2 is in line with our expectations and lower free energy of secondary structure of H1 and H2 indicates hairpins are more stable by NUPACK stimulation [33].

More importantly, we can calculate the equilibrium concentration for all of the structures in the thermodynamic limit of compound system, and it is important for designing and analyzing experiments. Consider the tube containing the set of strand species $\Psi_{0}$ that interacts to form a new set Ψ. The first step is to do the partition function Q calculation for each complex  j∈Ψ [41]. The equilibrium concentrations, $x_{\Psi }$, for the complexes are estimated by resolving the optimization problem in Equation (3) [42].

(4) $$\;\begin{array}{c} \;\min\\ x_{\Psi } \end{array}\underset{j\epsilon \Psi }{\sum }x_{j}\left(\log\;x_{j}-\log\;Q_{i}-1\right)\;\;\mathrm{subject}\;\mathrm{to}\;A_{i,j}x_{j}=x_{i}^{0}\;\forall \;i\in \Psi^{0}\;$$

Here, the constraints enforce conservation of mass. *A* stands for the stoichiometry matrix with entries $A_{i,j}$ corresponding to the quantity of complex of type *i* in complex *j*. $x_{i}^{0}$ is used to represent total concentration of complex *i*.

**Figure 3:**
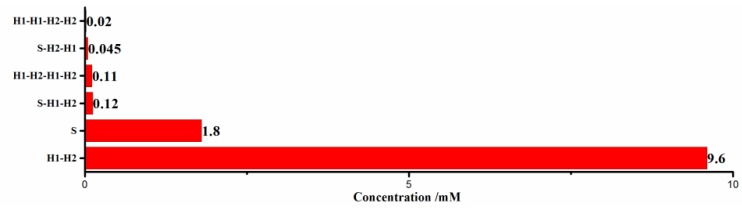
Simulation results of the catalyzed hairpin assembly (CHA) process in our strategy.

Based on the above theory and computation, the equilibrium concentration for each species of complex in CHA process of our strategy is shown in Figure 3. Multiple molecular structures appeared at equilibrium, and their proportion is in line with our expectations. For example, the concentration of H1–H2 strands is much more than that of by-products, such as S–H1–H2 strands, H1–H2–H1–H2 strands, and so on. The simulation results are encouraging, indicating that the sequence design of hairpins H1 and H2 is reasonable in our work, theoretically.

**Figure 4:**
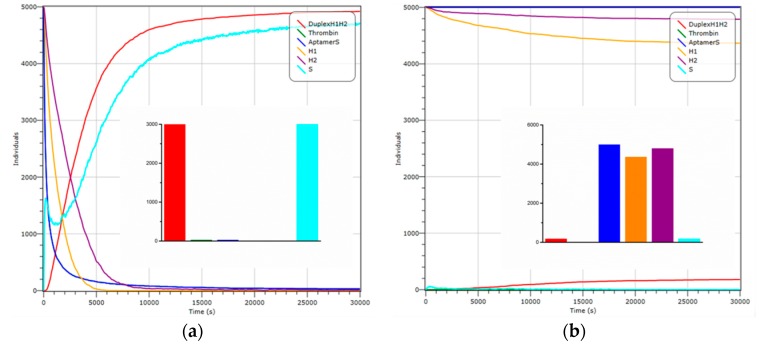
Visual DSD simulations. The change in the number of strands over time in the system with thrombin (**a**); without thrombin (**b**). Inset: the concentration of main strands at the equilibrium state corresponding to (**a**) and (**b**), respectively.

In addition, the process of reaction is simulated by Visual DSD [34] before biological testing, to verify the feasibility theoretically, and simplify the subsequent actual experiments (Figure 4). Here, we process the thrombin into a single strand for convenience, which is complementary to the thrombin aptamer. In the presence of thrombin, the substrate strands change over time in the system (Figure 4a). Since thrombin and Aptamer-S interact strongly, the concentration of thrombin and the Aptamer-S strands decreases rapidly, and tends to 0 ultimately (Figure 4a, green curve, blue curve, respectively). The liberated S strands increase firstly, and then fall sharply in the process of CHA, but increase to maximum value finally (Figure 4a, light blue curve). In this process, the hairpins H1 and H2 drop to a smaller value quickly (Figure 4a, yellow curve, purple curve, respectively), while the concentration of self-assembly products, DuplexH1H2, rise continuously, and maintain balance eventually (Figure 4a, red curve). On the contrary, in the absence of thrombin, the concentration change of control group is shown in Figure 4b. Both thrombin and S remain at the 0 value (Figure 4b, green curve, light blue curve), so the Aptamer-S strands remains in the initial state (Figure 4b, blue curve). Importantly, there is a small amount of DuplexH1H2 generated because of the weak interaction between H1 and H2 (Figure 4b, yellow curve, purple curve, red curve, respectively). The inset figures show the concentration of main strands at the equilibrium state in these two cases, respectively. As we can see, in the presence of thrombin, the value of DuplexH1H2 is about 30 times of that without thrombin. Therefore, the simulation results prove the feasibility of the detection method, theoretically. Beyond that, a series of reactions are simulated and measured with different conditions, roughly determining the range of concentration of each DNA strands initially in biological experiments.

### 3.3. Feasibility of CHA Amplification Approach for Thrombin Analysis

**Figure 5:**
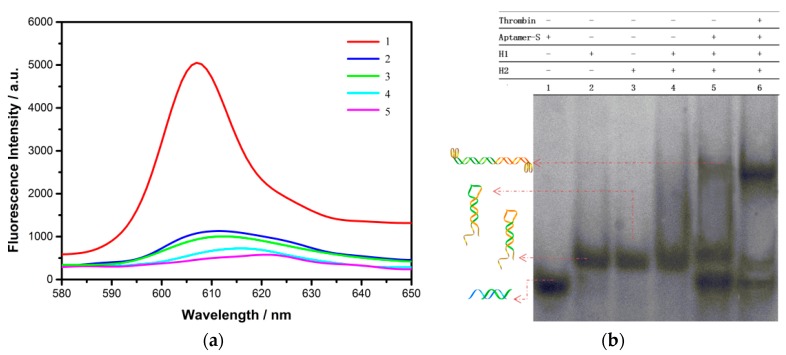
(**a**) Fluorescence spectra of NMM of different samples: (1) Aptamer-S, H1, H2, and thrombin; (2) Aptamer-S, H1 and H2; (3) H1 and H2; (4) Aptamer-S, thrombin; (5) Aptamer-S. The concentration of thrombin, Aptamer-S, H1 and H2 are 1 nM, 50 nM, 300 nM and 300 nM, respectively. (**b**) 10% native-PAGE analysis of different solutions: (1) Aptamer-S; (2) H1; (3) H2; (4) H1 and H2; (5) Aptamer-S, H1, and H2; (6) Aptamer-S, H1, H2, and thrombin. The concentration of thrombin, Aptamer-S, H1, and H2 are 1 μM, 5 μM, 5 μM, and 5 μM, respectively.

First of all, the fluorescence intensity changes of NMM were performed to prove the feasibility of this method for thrombin detection. Fluorescence signal was hardly observed from samples containing Aptamer-S (curve 5) in Figure 5a. Compared with curve 5, there was weak growth upon the addition of thrombin (curve 4), due to the aptamer strand being folded into a few G-quadruplexes, and led to the slight increase in the optical signal. In addition, the fluorescence intensity of test tubes containing the mixtures of H1, H2 (curve 3), and the mixtures of Aptamer-S, H1, H2 (curve 2) were slightly higher than that of curve 4 and 5, since weak interaction of hairpins produced a small proportion of G-quadruplexes at both ends. Then, the addition of target protein to the test tube containing Aptamer-S, H1, H2 strand, contributed to distinguishable signal enhancement (curve 1). Through the above analysis of the corresponding fluorescence intensity of different samples, it is proven that the reaction process is in agreement with our expectations, and the sensing model can be utilized to detect thrombin.

In addition, native PAGE had been applied to describe the dynamic process of molecular reactions in Figure 5b. The distinct bands from lane 1–3 corresponded to Aptamer-S, H1, and H2 strand, respectively. The mixture of H1 and H2 had similar bands with H1 or H2 (lane 4), which suggested almost no self-assembly reaction between H1 and H2. When Aptamer-S strand was added to the following system, there was a weak band above H1 and H2, because H1 and H2 mixture produced a negligible H1/H2 complex (lane 5). More importantly, in the presence of thrombin, a much brighter band corresponding to H1/H2 complex came into being in the Aptamer-S solution, followed by the addition of H1, H2 (lane 6). At the same time, by the function of thrombin, the band intensities of H1, H2 and Aptamer-S were growing weaker, giving adequate evidence to the fact that the hairpins were involved in the self-assembly process, and transformed into other products. The results are consistent with the fluorescence data, giving further verification on the feasibility of our sensing platform.

**Figure 6:**
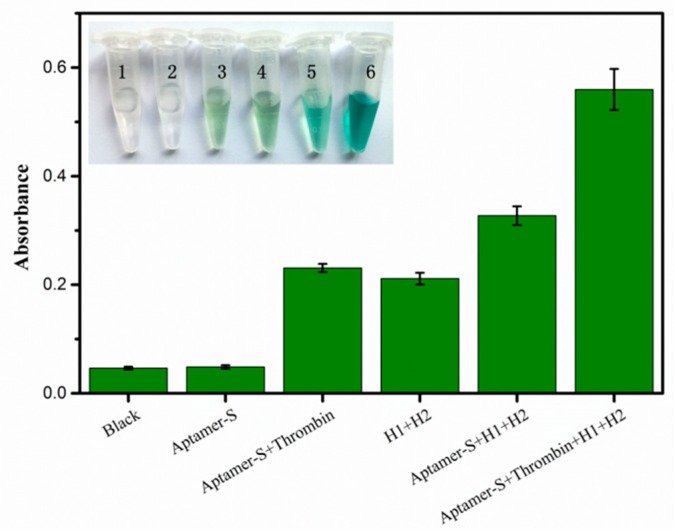
Absorption intensity value of TMB of different samples. Inset: the corresponding photographs of the colorimetric responses. The concentrations of thrombin, Aptamer-S, H1, and H2 are 1 nM, 50 nM, 300 nM, and 300 nM, respectively. Error bars show the standard deviation of three experiments.

Moreover, the strategy proposed in this work can visually detect thrombin by observing the color change of the solution, which is available for real-time diagnosis and monitoring of illnesses. In this case, G-quadruplex/hemin DNAzyme that mimicked peroxidase activities was employed in the experiments, and catalyzed the oxidation of TMB, by H_2_O_2_, to a green-colored end-product. The existence of thrombin would have a considerable effect on the solution reaction in accordance with same principle. As shown in Figure 6, the solution with Aptamer-S, thrombin, H1, and H2 emitted the highest optical signal, and different samples would be identified by observing the color change visible to the naked eye (inset of Figure 6). In addition, the unspecific signals were not to be ignored, although they could be distinguished from specific signals. Prevention of non-target-trigger reaction by further optimizing the design of the hairpin sequences may become crucial in follow-up studies.

### 3.4. Optimization of Detection Condition

**Figure 7:**
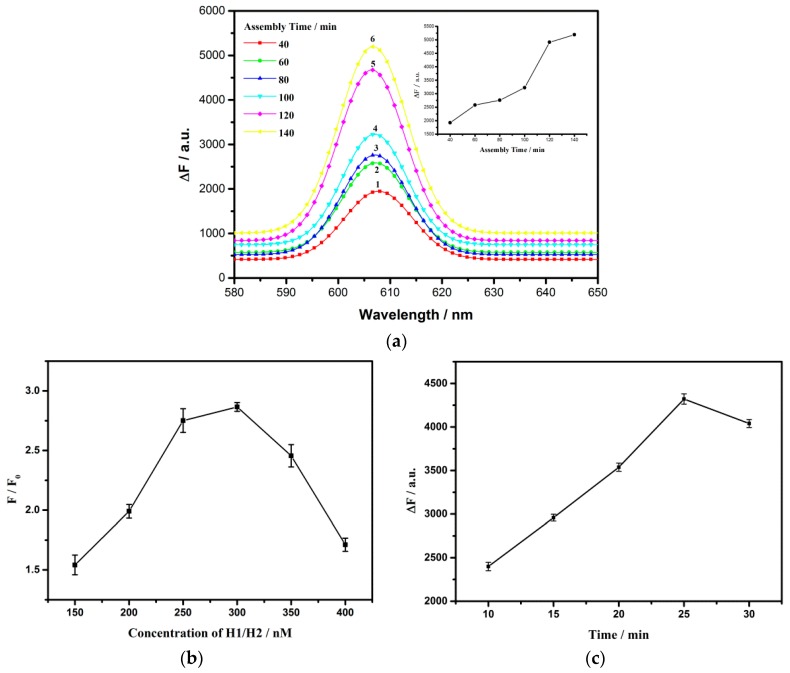
(**a**) The change in fluorescence emission spectra to different assembly time. Inset: the change in fluorescence emission intensity at 608 nm from NMM vs the assembly time; (**b**) The change in fluorescence emission spectra to the reaction time between G-quadruplex structure and NMM; (**c**) The change in fluorescence emission spectra to the concentration of H1 and H2. Error bars show the standard deviation of three experiments.

To achieve the optimum analysis performance of this platform, the incubation time between G-quadruplex and NMM, the reaction time, and the concentration of hairpins were researched. The fluorescence intensity of the mixed solution is indicated by F, while the blank is F_0_, and ∆F represents the amount of fluorescence change. The reaction time of CHA was investigated, and Figure 7a displays the change in fluorescence emission signals corresponding to a series of different assembly time. What we can infer from this is that the ∆F value keeps growing when the assembly time is elevated from 40 min to 140 min. By presenting the relationship between fluorescence intensity at 609 nm and the assembly time (insert of Figure 7a), ΔF increases with reaction time of hairpins, and then remains steady after 120 min. Therefore, the optimal reaction time for CHA was set to 120 min in our experiments. In addition, the fluorescence intensity also depended directly on the interaction time between G-quadruplex structures and NMM. According to Figure 7b, one can see that ∆F reached the peak when the interaction time was 25 min, indicating that 25 min was a judicious choice for the interaction. Under given condition, the amount of G-quadruplexes was related to the concentration of H1/H2, deciding the intensity of the fluorescence, finally. As shown in Figure 7c, the signal-to-noise ratio (F/F0) promptly increased along with increasing of H1 and H2 concentration in the range from 150 to 300 nM, and then gradually decreased thereafter. With the increase of hairpin concentration, the noise caused by non-specific reactions had an inevitable increase as well, while the rate and yield of assembly enhanced. Based on the above reason, 300 nM was selected as the optimal concentration of H1 and H2.

### 3.5. Sensitive Detection of Thrombin with Fluorescence as Signal Report

**Figure 8:**
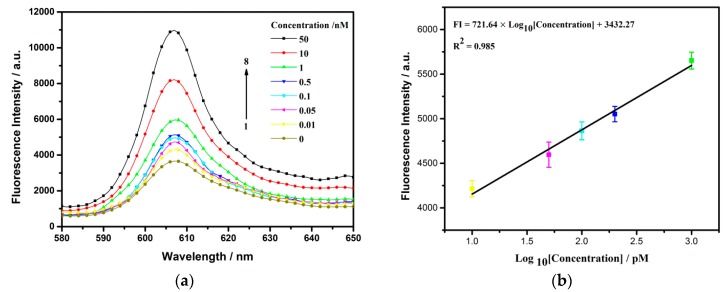
(**a**) Florescence spectra of NMM with different concentrations of thrombin. From curve 1 to 8, the concentration of thrombin is 0, 0.01, 0.05, 0.1, 0.5, 1, 10, 50 nM, respectively; (**b**) Liner correlation of the fluorescence change vs logarithmic concentration of thrombin. The standard errors of slope and intercept are 43.39 and 91.40, respectively. Error bars show the standard deviation of three experiments.

Sensitive is critical for early and accurate detection, and considered as an important index reflecting biosensor performance. A series of concentrations of thrombin were measured under the optimum operation conditions. As can be seen from Figure 8a, the fluorescence of NMM gradually enhanced with the increasing of thrombin concentration from 0.01 nM to 50 nM. Benefitting from the amplified method, the relation between fluorescence intensity at 608 nm and logarithm of the thrombin concentration from 0.01 nM to 1 nM, displayed a positive relativity (Figure 8b). The linear regression equation could be described as follows: *Y* = 721.64 lg [*X*] + 3432.27 with a correlation coefficient of 0.985. *Y* and *X* denoted to the fluorescence intensity and the concentration of thrombin. Additionally, the limit of detection (LOD) for thrombin was calculated to be 2.4 pM (3σ/S, in which σ is the standard deviation for the blank solution, and S is the slope of the linear equation). Compared to some previous optical strategies, our approach not only held the feature of lower detection limit, but also was more convenient, due to the non-label and enzyme-free design (Table 2).

### 3.6. Detection of Thrombin in Bovine Serum

**Figure 9:**
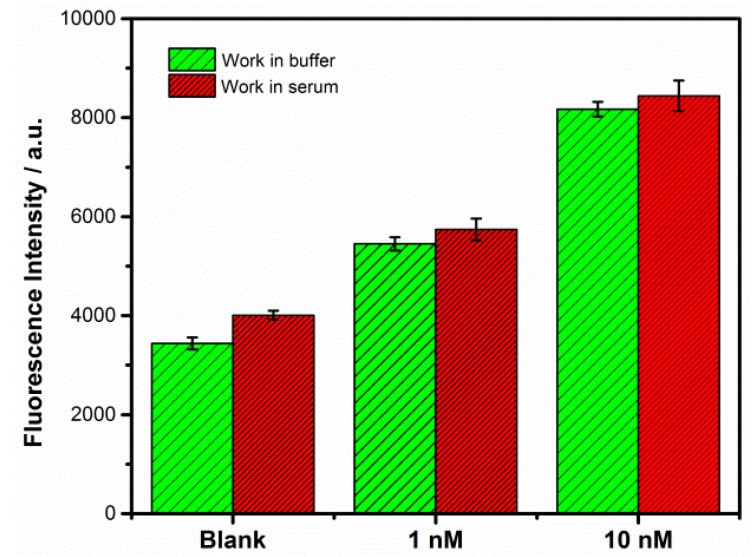
Results obtained from the fluorescent tests of Tris–HCl buffer and diluted serum samples spiked with different concentrations of thrombin. The same reaction mixtures without thrombin were used as blank. Error bars show the standard deviation of three experiments.

To verify the feasibility of the strategy in the biological environment, the sensing platform was employed to detect the target thrombin in bovine serum samples. As shown in Figure 9, compared with blank samples, fluorescence enhancement signal could be seen for thrombin detection in both buffer solution and serum. In addition to weak interaction of hairpins, relatively high background noise may be due to the formation of G-quadruplexes induced by potassium ions in serum. The result indicates that the proposed method has the potential application for thrombin detection in real biological samples.

## 4. Conclusions

In summary, this paper has presented a non-label and enzyme-free fluorescent/colorimetric approach for thrombin detection, demonstrated by both simulations and biological experiments. Our approach shares several distinct advantages, as follows. First, one characteristic of the sensing model has good capability of thrombin detection with no fluorescent modification, making the detection method more facile, economical, and effective. Second, the reaction process is verified and optimized initially by simulations before attempting to build them in lab, to ensure that the actual experiments are more efficient. Third, this fluorescent strategy allows the enlargement of signal without the involvement of other proteases and complicated thermal cycling process. This biosensor based on CHA signal amplification can sensitively detect thrombin down to 2.4 pM. At the same time, micro analytical reagent is clearly distinguished by the naked eye, without instrumentation and accessories, in a colorimetric assay. Finally, by combining biological calculations with biological reactions in living organisms, the proposed sensing platform we designed has been proven with regard to the theoretical feasibility, verified simultaneously by experiments with various kinds of biological experiment methods. Hence, the platform has a fairly good universal adaptability for detection of other targets by ingenious design probe sequence, and certain potential applications in biomarker detection and a quantitative monitor for disease diagnosis. Taken together, our strategy has good potential in applications such as the interdisciplinary subjects of computer and biology, which provides new ideas for future biological research.

## Figures and Tables

**Table 1:** Sequences of oligonucleotides.

Name	Sequences (5′–3′)
Aptamer	GGT TGG TGT GGT TGG AAT AGT C
S	AGT CAC ACG GAC TAT TCC AA CC
H1	GGG TAA TAG TCC GTG TGA CTA TGG ACT ATA GGA GTC ACA CGG GCG GGT AGG G
H2	GGG TTG TAT GAC TCC TAT AGT CCA TAG TCA CAC GGA CTA TTG GGC GGG TAG GG

**Table 2:** Comparison of the methods with the previous fluorometric/colorimetric for thrombin detection.

Analytical Method	Dynamic Range	Detection Limit	References
Fluorescence detection	0–500 nM	1.1 nM	[5]
Fluorescence detection	0.02–2 nM	20 pM	[7]
Fluorescence detection	0–1 nM	100 pM	[8]
Colorimetric detection	2.5–2500 pM	1.9 pM	[29]
Fluorescence detection	0.01–50 nM	2.4 pM	Our method

## References

[1] Lowe C.R. (1985). As introduction to the concepts and technology of biosensors. Biosensors.

[2] Scheller F.W., Hintsche R., Pfeiffer D., Schubert F., Riedel K., Kindervater R. (1991). Biosensors: Fundamentals, applications and trends. Sens. Actuators B Chem..

[3] Tan Y., Guo Q., Zhao X., Yang X., Wang K., Huang J., Zhou Y. (2014). Proximity-dependent protein detection based on enzyme-assisted fluorescence signal amplification. Biosens. Bioelectron..

[4] Becker R.C., Spencer F.A. (1998). Thrombin: Structure, Biochemistry, Measurement, and Status in Clinical Medicine. J. Thromb. Thrombolysis.

[5] Kong L., Xu J., Xu Y., Xiang Y., Yuan R., Chai Y. (2013). A universal and label-free aptasensor for fluorescent detection of ATP and thrombin based on SYBR Green I dye. Biosens. Bioelectron..

[6] Tan Y., Zhang X., Xie Y., Zhao R., Tan C., Jiang Y. (2012). Label-free fluorescent assays based on aptamer-target recognition. Analyst.

[7] Zheng A.X., Wang J.R., Li J., Song X.R., Chen G.N., Yang H.H. (2012). Enzyme-free fluorescence aptasensor for amplification detection of human thrombin via target-catalyzed hairpin assembly. Biosens. Bioelectron..

[8] Xue L., Zhou X., Da X. (2012). Sensitive and Homogeneous Protein Detection Based on Target-Triggered Aptamer Hairpin Switch and Nicking Enzyme Assisted Fluorescence Signal Amplification. Anal. Chem..

[9] Xue L., Zhou X., Xing D. (2010). Highly sensitive protein detection based on aptamer probe and isothermal nicking enzyme assisted fluorescence signal amplification. Chem. Commun..

[10] Niu S., Qu L., Zhang Q., Lin J. (2012). Fluorescence detection of thrombin using autocatalytic strand displacement cycle reaction and a dual-aptamer DNA sandwich assay. Anal. Biochem..

[11] Assicot M., Bohuon C. (1971). Presence of two distinct catechol-O-methyltransferase activities in red blood cells. Biochimie.

[12] Lequin R.M. (2005). Enzyme Immunoassay (EIA)/Enzyme-Linked Immunosorbent Assay (ELISA). Clin. Chem..

[13] Lam M.T., Wan Q.H., Boulet C.A., Le X.C. (1999). Competitive immunoassay for staphylococcal enterotoxin A using capillary electrophoresis with laser-induced fluorescence detection. J. Chromatogr. A.

[14] Baker K.N., Rendall M.H., Patel A., Boyd P., Hoare M., Freedman R.B., James D.C. (2002). Rapid monitoring of recombinant protein products: A comparison of current technologies. Trends Biotechnol..

[15] Yin P., Choi H.M., Calvert C.R., Pierce N.A. (2008). Programming biomolecular self-assembly pathways. Nature.

[16] Choi M.S., Yoon M., Baeg J.O., Kim J. (2009). Label-free dual assay of DNA sequences and potassium ions using an aptamer probe and a molecular light switch complex. Chem. Commun..

[17] Fu B., Cao J., Jiang W., Wang L. (2013). A novel enzyme-free and label-free fluorescence aptasensor for amplified detection of adenosine. Biosens. Bioelectron..

[18] Li J., Macdonald J. (2015). Advances in isothermal amplification: Novel strategies inspired by biological processes. Biosens. Bioelectron..

[19] Xu Y., Zhou W., Zhou M., Xiang Y., Yuan R., Chai Y. (2015). Toehold strand displacement-driven assembly of G-quadruplex DNA for enzyme-free and non-label sensitive fluorescent detection of thrombin. Biosens. Bioelectron..

[20] Lv L., Guo Z., Wang J., Wang E. (2012). G-quadruplex as signal transducer for biorecognition events. Curr. Pharm. Des..

[21] Lu L., Wang W., Wang M., Kang T.S., Lu J.J., Chen X.P., Han Q.B., Leung C.H., Ma D.L. (2016). A luminescent G-quadruplex-selective iridium(III) complex for the label-free detection of lysozyme. J. Mater. Chem. B.

[22] Lu L., Mao Z., Kang T.S., Leung C.H., Ma D.L. (2016). A versatile nanomachine for the sensitive detection of platelet-derived growth factor-BB utilizing a G-quadruplex-selective iridium(III) complex. Biosens. Bioelectron..

[23] Yan S., Huang R., Zhou Y., Zhang M., Deng M., Wang X., Yan S. (2011). Aptamer-based turn-on fluorescent four-branched quaternary ammonium pyrazine probe for selective thrombin detection. Chem. Commun..

[24] Bai D., Ji D., Shang J., Hu Y., Gao J., Lin Z., Ge J., Li Z. (2017). A rapid biosensor for highly sensitive protein detection based on G-quadruplex-Thioflavin T complex and terminal protection of small molecule-linked DNA. Sens. Actuators B Chem..

[25] Li H., Ren J., Liu Y., Wang E. (2014). Application of DNA machine in amplified DNA detection. Chem. Commun..

[26] Wang K., Ren J., Fan D., Liu Y., Wang E. (2014). Integration of graphene oxide and DNA as a universal platform for multiple arithmetic logic units. Chem. Commun..

[27] Li J., Jia Y., Zheng J., Zhong W., Shen G., Yang R., Tan W. (2013). Aptamer degradation inhibition combined with DNAzyme cascade-based signal amplification for colorimetric detection of proteins. Chem. Commun..

[28] Li W., Liu Z., Lin H., Nie Z., Chen J., Xu X., Yao S. (2010). Label-Free Colorimetric Assay for Methyltransferase Activity Based on a Novel Methylation-Responsive DNAzyme Strategy. Anal. Chem..

[29] Wu H., Zhang K., Liu Y., Wang H., Wu J., Zhu F., Zou P. (2015). Binding-induced and label-free colorimetric method for protein detection based on autonomous assembly of hemin/G-quadruplex DNAzyme amplification strategy. Biosens. Bioelectron..

[30] Kosman J., Juskowiak B. (2011). Peroxidase-mimicking DNAzymes for biosensing applications: A review. Anal. Chim. Acta.

[31] Zhang L., Zhu J., Li T., Wang E. (2011). Bifunctional colorimetric oligonucleotide probe based on a G-quadruplex DNAzyme molecular beacon. Anal. Chem..

[32] McCaskill J.S. (1990). The equilibrium partition function and base pair binding probabilities for RNA secondary structure. Biopolymers.

[33] Dirks R.M., Bois J.S., Schaeffer J.M., Winfree E., Pierce N.A. (2007). Thermodynamic Analysis of Interacting Nucleic Acid Strands. Siam Rev..

[34] Lakin M.R., Simon Y., Filippo P., Stephen E., Andrew P. (2011). Visual DSD: a design and analysis tool for DNA strand displacement systems. Bioinformatics.

[35] Bock L.C., Griffin L.C., Latham J.A., Vermaas E.H., Toole J.J. (1992). Selection of single-stranded-dna molecules that bind and inhibit human thrombin nature. Nature.

[36] Lee C.Y., Jang H., Park K.S., Park H.G. (2017). A label-free and enzyme-free signal amplification strategy for a sensitive RNase H activity assay. Nanoscale.

[37] Tinoco I., Borer P.N., Dengler B., Levine M.D., Uhlenbeck O.C., Crothers D.M., Gralla J. (1971). Improved Estimation of Secondary Structure in Ribonucleic Acids. Nature.

[38] Waterman M.S. (1978). Secondary Structure of Single-Stranded Nucleic Acidst. Stud. Found. Comb. Adv. Math. Suppl. Stud..

[39] Dirks R.M., Pierce N.A. (2008). An algorithm for computing nucleic acid base-pairing probabilities including pseudoknots. J. Comput. Chem..

[40] Dirks R.M., Pierce N.A. (2003). A partition function algorithm for nucleic acid secondary structure including pseudoknots. J. Comput. Chem..

[41] Wolfe B.R., Pierce N.A. (2015). Sequence Design for a Test Tube of Interacting Nucleic Acid Strands. ACS Synthetic Biol..

[42] Gallian J.A. (1998). Contemporary Abstract Algebra.

